# Retraction Note: Correlation of CD44v6 expression with ovarian cancer progression and recurrence

**DOI:** 10.1186/s12885-020-06753-0

**Published:** 2020-03-19

**Authors:** Jun Shi, Zhou Zhou, Wen Di, Ningli Li

**Affiliations:** 1 Shanghai Institute of Immunology, Shanghai Jiao Tong University School of Medicine, Shanghai, 200025 People’s Republic of China; 2 Department of Obstetrics and Gynecology, Ren ji Hospital, School of Medicine, Shanghai Jiao Tong University, Shanghai, 200127 People’s Republic of China

**Retraction Note: BMC Cancer (2013) 13: 182**

**https://doi.org/10.1186/1471-2407-13-182**

The authors have retracted this original article [1] because a number of the figures exhibit irregularities.

Specifically:

- Figure 1C appears to be comprised of lanes from multiple gels, with only one loading control. Further, the CD44v6 lanes seem to be rotated 180 degrees.
- In Figure 3, panels C and F overlap extensively.
- In Figure 5B, panels A and B overlap extensively.
- In Figure 5C, panels C and D overlap extensively. Also, panel D appears to be rotated 90 degrees.

As these irregularities undermine the conclusions presented, the Editor no longer has confidence in the article.

All authors agree with this retraction.
